# Investigation on Inter-Limb Coordination and Motion Stability, Intensity and Complexity of Trunk and Limbs during Hands-Knees Crawling in Human Adults

**DOI:** 10.3390/s17040692

**Published:** 2017-03-28

**Authors:** Shenglan Ma, Xiang Chen, Shuai Cao, Yi Yu, Xu Zhang

**Affiliations:** Department of Electronic Science and Technology, University of Science and Technology of China, Hefei 230026, China; msl92072@mail.ustc.edu.cn (S.M.); caoshuai@ustc.edu.cn (S.C.); yyu309@mail.ustc.edu.cn (Y.Y.); xuzhang90@ustc.edu.cn (X.Z.)

**Keywords:** hands-knees crawling, accelerometer, inter-limb coordination, motion stability, motion complexity

## Abstract

This study aimed to investigate the inter-limb coordination pattern and the stability, intensity, and complexity of the trunk and limbs motions in human crawling under different speeds. Thirty healthy human adults finished hands-knees crawling trials on a treadmill at six different speeds (from 1 km/h to 2.5 km/h). A home-made multi-channel acquisition system consisting of five 3-axis accelerometers (ACC) and four force sensors was used for the data collection. Ipsilateral phase lag was used to represent inter-limb coordination pattern during crawling and power, harmonic ratio, and sample entropy of acceleration signals were adopted to depict the motion intensity, stability, and complexity of trunk and limbs respectively. Our results revealed some relationships between inter-limb coordination patterns and the stability and complexity of trunk movement. Trot-like crawling pattern was found to be the most stable and regular one at low speed in the view of trunk movement, and no-limb-pairing pattern showed the lowest stability and the greatest complexity at high speed. These relationships could be used to explain why subjects tended to avoid no-limb-pairing pattern when speed was over 2 km/h no matter which coordination type they used at low speeds. This also provided the evidence that the central nervous system (CNS) chose a stable inter-limb coordination pattern to keep the body safe and avoid tumbling. Although considerable progress has been made in the study of four-limb locomotion, much less is known about the reasons for the variety of inter-limb coordination. The research results of the exploration on the inter-limb coordination pattern choice during crawling from the standpoint of the motion stability, intensity, and complexity of trunk and limbs sheds light on the underlying motor control strategy of the human CNS and has important significance in the fields of clinical diagnosis, rehabilitation engineering, and kinematics research.

## 1. Introduction

Normal crawling is often regarded as a sign of the normal development of infants and young children [1]. Crawling can be divided into hand-knee type, hand-foot type, scooting type, creeping type, and mixed type, etc. [2]. Toddlers crawl before they can walk unsupported, and most of them crawl via the hand-knee type [3]. In clinic, the development situation of infants and young children can be evaluated by analyzing their crawling function. In the field of cerebral palsy (CP) rehabilitation [4,5], crawling is an excellent training item because it requires the patients to coordinate their multiple body parts and utilize audio and visual aids as their motion guide. In the field of neuroscience, crawling is a good research object to explore the underlying mechanisms of inter-limb coordination in quadrupedal locomotion as it is a common locomotion in both human and animals [6,7,8].

The study of inter-limb coordination during crawling in both human and animals has attracted certain amount of attention in the twentieth and twenty first centuries. Early researches based on video records found that there were various inter-limb coordination patterns in crawling [9,10]. In these studies, some subjects were found to crawl with diagonal limbs moving together, some subjects crawled with ipsilateral limbs moving together, and others did not belong to either pattern, as their four limbs moved in turn during crawling. Hildebrand first presented that four-limb locomotion could be divided into different patterns based on the “percent of stride interval that footfall of forefoot follows hind on same side” [11], and Patrick et al. named it as ipsilateral phase lag (IPL) in 2009 [6]. IPL, which is the phase lag between stance of the ipsilateral arm and leg, can be sufficient to quantify the inter-limb coordination patterns. IPL values closing to 50% indicate that diagonal limbs enter stance phase together. IPL values closing to 0% or 100% describe that ipsilateral limbs enter stance phase together, and values like 25% or 75% mean that four limbs entered stance phase alternately. Based on IPL, a number of studies have been conducted to explore the similarities and differences of inter-limb coordination between animals, human infants, and human adults. For instance, Courtine et al. compared four-limb locomotion of a rhesus (non-human primate) with sub-primate quadrupedal mammals [12]. Patrick et al. compared the inter-limb coordination across human adults, infants, and quadrupeds during four-limb locomotion [6]. They found that, unlike the lateral sequence characterizing in sub-primate, primates tended to use diagonal coordination between hind-limbs and fore-limbs, more similar to that always observed in human gaits between legs and arms [13,14,15,16,17,18]. Besides, crawling inter-limb coordination patterns have been found to change during the growth and development of human beings. Due to the immaturity of the nervous system, most infants only used diagonal coordination, while adults had greater flexibility in coordinating limbs [12].

Meanwhile, studies showed that changes in speed, inclination, midline obstruction, and hind-limb length could affect inter-limb coordination during crawling movement [3,6,19,20]. Typically, human adults showed high flexibility in crawling with changing speeds. When crawling speed increased, cycle duration decreased and the proportion of swing time increased [3]. In addition, cycle duration did not show a difference between the upper and lower limbs, and the swing duration was consistently shorter in the upper limbs compared with the lower limbs [3,21]. Compared with the high speed, human adults showed a greater occurrence of IPL value around 25% (no limb pairing) at low speed. Besides, Sparrow et al. noticed that there was an abrupt change of inter-limb coordination pattern in hand-foot crawling at about 50% of maximum speed [22]. However, MacLellan et al. found that patterns did not change a lot during hand-foot crawling [3]. Meanwhile, adults exhibited a great variety of inter-limb coordination patterns during crawling with changing speeds, but there was a weak relationship between speeds and inter-limb coordination patterns, as the selection of coordination patterns did not show a linear or consistent relationship with crawling speed [3].

Although considerable progress has been made in the study of four-limb locomotion, much less is known about the reason for the variety of inter-limb coordination. So far, there are three speculations about the inter-limb coordination pattern change under changing speeds. One suggestion is that pattern changes are related to aerobic energy expenditure [23]. Another suggestion is that the changes are related to storage of elastic energy [24]. The third suggestion is that physical constraints of the organism influence the emergence of new movement patterns, such as the limb’s ability to produce force and physical properties of the limb such as mass and length [22]. These speculations have not yet been confirmed with enough evidences.

Taking human adults and hands-knees crawling as research objects, this study aimed to investigate the inter-limb coordination pattern choice under different speeds from aspects of the motion stability, intensity and complexity of trunk and limbs. In this study, crawling motion information was captured by two kinds of sensors including accelerometers and force sensors. Based on the analysis of the motion stability, intensity, and complexity of three inter-limb coordination patterns (trot-like, pace-like, and no-limb-pairing), the possible reasons for the inter-limb coordination choice under different crawling speeds were explored. The results of this study showed that the CNS tended to choose a stable inter-limb coordination pattern to ensure the safety of the body and avoid falling. This exploration can help us to understand the underlying motor control strategy of the human CNS and has important significance in the fields of clinical diagnosis, rehabilitation engineering, and kinematics research. 

## 2. Materials and Methods

### 2.1. Subjects and Crawling Scheme

30 healthy adults (19 males and 11 females, 23.57 ± 0.86 year of age (mean ± SD); females: height: 161.64 ± 3.67 cm, weight: 47.36 ± 3.70 kg; males: height: 173.37 ± 5.00 cm, weight: 67.11 ± 7.45 kg) were recruited in this study. All volunteers had no history of joints injuries or neuromuscular disease. All subjects were informed of the experiment procedure and signed an informed consent approved by the local Ethics Review Committee. This study was conducted in accordance with the Helsinki Declaration.

In this study, a treadmill (F63 PRO, SOLE, West Hollywood, CA, America, belt size: 510 mm × 1550 mm, treadmill size: 204 cm × 89 cm × 145 cm, speed range: 1–18 km/h, incline range: 0°–15°) was used as the crawling platform. During experiment, all subjects were instructed to crawl with hands and knees on the treadmill with 0° inclination at speeds of 1, 1.3, 1.5, 1.8, and 2 km/h (range from the slowest possible speed for the treadmill to the highest speed that all subjects could achieve). In addition, 21 of 30 participants finished crawling task at the speed over 2 km/h. The maximal speed was chosen to be 2.5 km/h for safety consideration. Each subject finished at least 20 consecutive strides under each speed, and there was a rest period of 1 minute between two trials to avoid fatigue. 

### 2.2. Crawling Data Acquisition Based on Accelerometers and Force Sensors

**Figure 1:**
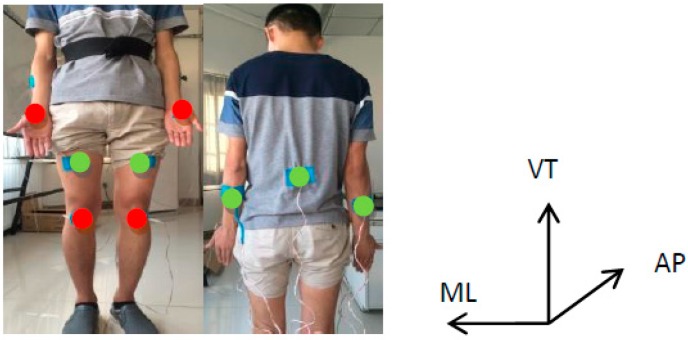
The placement of force sensors and accelerometers. Red circles represent force sensors, and green circles represent accelerometers. Each accelerometer was arranged to measure the dynamic accelerations in the directions of anterior-posterior (AP), vertical (VT), and medio-lateral (ML) synchronously.

In order to capture the movements of four limbs and trunk in hands-knees crawling effectively, a home-made multi-channel acquisition system consisting of five 3-axis accelerometers (ACC) and four force sensors was used for the data collection in this study. To capture the movements of fore- and hind-limbs effectively, as shown in Figure 1, two accelerometers were positioned bilaterally over the middle point of the outside of forearms and two accelerometers were placed over the middle point of the front side of thigh bilaterally. One accelerometer was placed on the waist to describe the movements of the trunk. Four force sensors were placed on the four points contacting with the ground during crawling. Specifically, two force sensors were positioned on the thenar of left and right hands, and the other two were placed below knees. All sensors were fixed by muscle stickers. Accelerometers produced digitalized data with 100 Hz sampling rate for each axis. The sampling rate of force sensor was also 100 Hz. Two kinds of signals were collected synchronously at the same sampling frequency. All the recorded data were wirelessly transmitted to a laptop via Bluetooth and saved to the laptop disk for offline analysis. All data analysis was finished in Matlab environment (version R2014a, The MathworksInc, Natick, MA, USA).

### 2.3. Crawling Data Analysis

**Figure 2:**
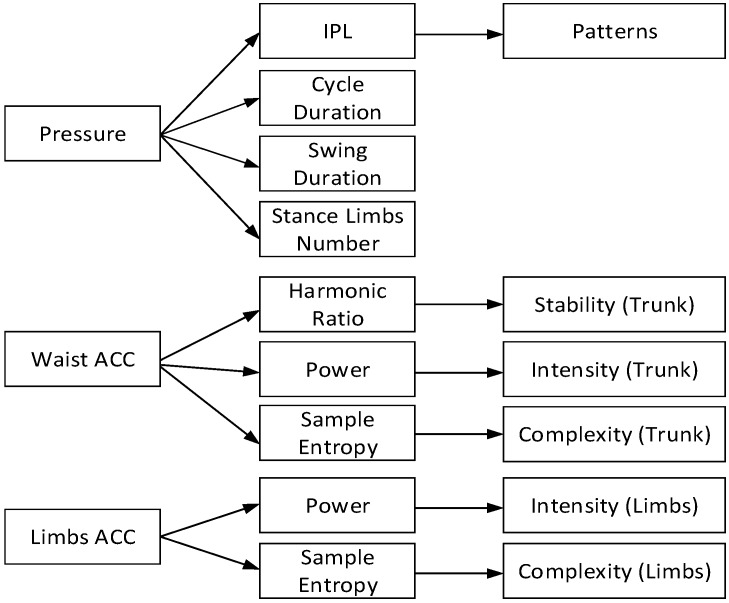
The flow chart of crawling data analysis.

As shown in the flow chart of crawling data analysis (Figure 2), pressure signals were used to obtain swing phase, stance phase, and cycle duration of crawling strides, and classify inter-limb coordination patterns by calculating IPL. Harmonic ratio, power, and sample entropy of waist ACC signals were calculated to measure the stability, intensity and complexity of trunk movement respectively. Power and sample entropy of limbs’ ACC were used to depict the intensity and complexity of limbs’ movements.

#### 2.3.1. Data Pre-Processing and Stride Segmentation

At the preprocessing stage, the mean values of acceleration data were subtracted to reduce the individual difference. Then, the acceleration data was low-pass filtered with cutoff of 20 Hz to eliminate high-frequency noise, because most acceleration energy focused on 0–15 Hz according to Karantonis’s studies [25]. To avoid the instability caused by onset and offset of locomotion, the first and last several strides data in each trial was omitted, and 15 consecutive strides data without any perturbations was analyzed, as 10 strides were sufficient for analysis according to Patrick’s studies [6]. 

**Figure 3:**
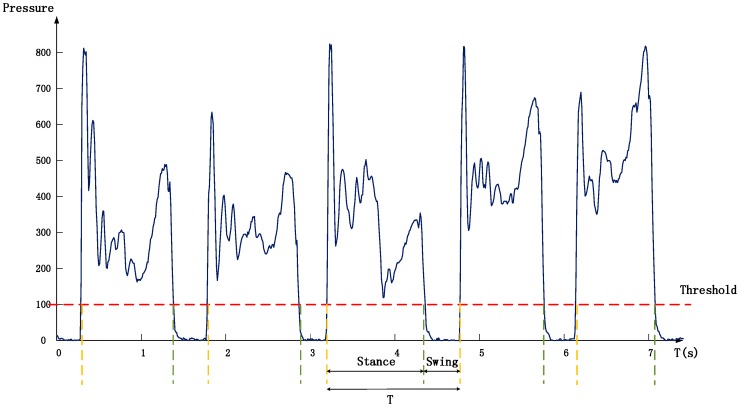
Stance and swing determined by pressure signals from the left knee. A stride cycle begins when the left knee strikes ground and ends when the next left knee striking happens. (Pressure sensor: FSR402, Interlink Electronics, Camarillo, CA, USA).

Before further analysis, continuous signals were segmented to obtain crawling cycle data. In this study, the initiation of swing phase of one limb was defined to be the point that the limb lift off from the belt, and the initiation of stance phase was the point that the limb contacted with the belt. As shown in Figure 3, stance and swing of limbs were determined from the pressure data by setting a threshold. Periods with pressure values over the threshold corresponded to stance phase. Otherwise, periods belonged to the swing phase. In this study, we defined that the crawling cycle began when the left knee struck ground and ended when the next left knee strike happened. So, two initiation points of the stance phase of left knee were detected to segment stride signals.

#### 2.3.2. Crawling Movement Features Extraction

● Inter-limb coordination parameter

**Figure 4:**
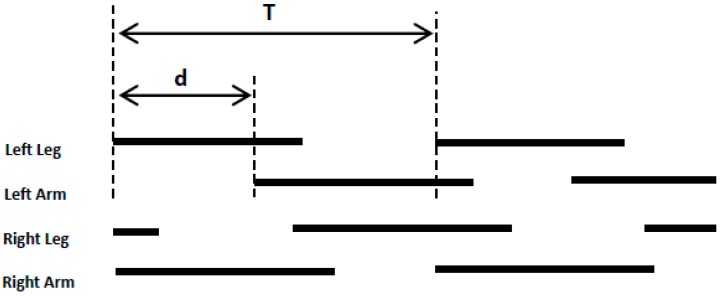
Illustration of ipsilateral phase lag (IPL) with limbs contact pattern [6]. Stance showed by solid lines and swing showed by space.

The movement of each limb consists of swing phase and stance phase during a crawling cycle. In this study, ipsilateral phase lag (IPL) was used to quantify the inter-limb coordination pattern during crawling movement. Using the method presented by Susan K Patrick et al. (2009), IPL can be expressed as Figure 4 and Formula (1)

(1) $$\mathrm{IPL}=\frac{d}{T}\times 100\%$$

where “*d*” is a stance phase delay in ipsilateral limbs, for example, the interval time between the initiation of stance phase in the left hand and left knee, and “*T*” represents a crawling cycle duration. 

According to the value of IPL, quadruped crawling can be classified into three inter-limb coordination patterns: (1) pace-like crawling, where IPL values are close to 0% or 100%, which means ipsilateral limbs enter stance phase together; (2) trot-like crawling, where IPL values are close to 50%, which indicates that diagonal limbs enter stance at the same time nearly; and (3) no limb pairing crawling, where IPL values are around 25% or 75%, which means four limbs enter stance phase in turns during a stride cycle.

● Trunk stability parameter

Crawling is a cyclical movement. During crawling movement, the trunk moves periodically, and the repeatability of this pattern can be used to judge the smoothness of the movement. According to H. John Yack and Roseanne C. Berger [26], comparing the “in phase” parts of movement with the “out of phase” parts by relatively magnitudes of the harmonic coefficients for the first 20 harmonics can describe the smoothness of cyclic motion. Some researches indicated that the waist ACC signals in VT and AP orientation were biphasic, so the value of harmonic ratio in VT and AP orientation was greater than 1. Meanwhile, the waist ACC signal in medio-lateral (ML) orientation was monophasic, and the value of harmonic ratio in ML orientation was less than 1 [22,27,28]. Besides, the stride pattern is smoother, the harmonic ratio is larger. Therefore, HR of waist ACC in ML, VT, and AP orientation was adopted to describe the trunk stability during crawling in this study.

When the Fourier transform of a segmented ACC data was calculated as Formula (2), harmonics ratio could be computed according to Formula (3).

(2) $$acc_{stride}=\overset{N-1}{\underset{n=0}{\sum }}C_{n}\sin\left(n\omega_{0}t+\varphi_{n}\right)$$

In Formula (2), $C_{n}$ is the harmonic coefficient, $\omega_{0}$ is the frequency, and $\varphi_{n}$ is the phase.

(3) $$\mathrm{HR}=\frac{\sum_{n=2,4,6,\ldots }^{20}C_{n}}{\sum_{n=1,3,5,\ldots }^{19}C_{n}}$$

● Movement intensity parameter

Power is a physical parameter that can measure the intensity of the movement. In this study, power values were calculated as the integral of the power spectral density of the waist or limbs ACC signal first, and then were log transformed to obtain a normal distribution as Formula (4).

(4) $$\mathrm{power}=\log\left(\int^{\text{​}}PSD\left(acc\right)\right)$$

The power parameter can capture the variations of amplitude and frequency movement during crawling, though motion frequency has a larger influence on the power value. It can also reflect the energy profile of movement [29]. The movement is more intense, the value of power is higher.

● Movement complexity parameter

Sample entropy is a parameter to measure time series complexity of signals [30,31], and larger sample entropy indicates higher complexity. In this study, sample entropy values of ACC signals of waist and limbs were calculated with the method introduced by Richman et al. [30] to describe the movement complexity of trunk and limbs. 

## 3. Results

### 3.1. Inter-Limb Coordination Variations under Different Crawling Speeds

**Figure 5:**
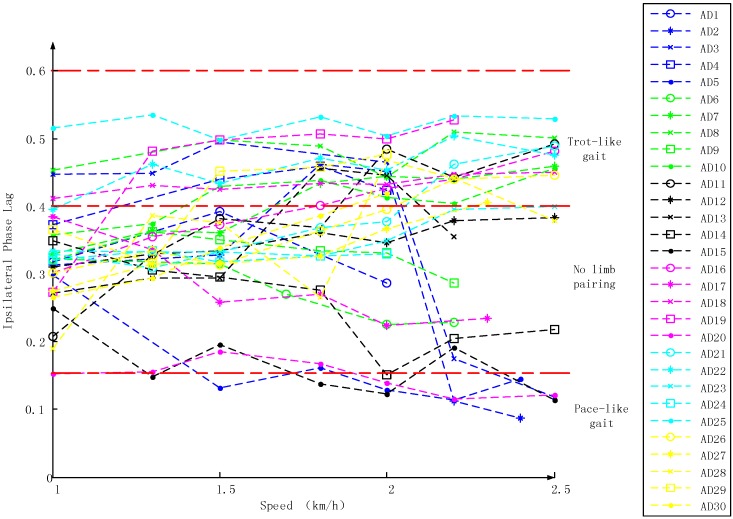
IPL values versus crawling speeds. Data from all 30 subjects and each line corresponds to one subject.

In order to show inter-limb coordination pattern choice at different crawling speeds, Figure 5 gave 30 subjects’ IPL values obtained at different crawling speeds. From the graph, it can be seen that adults show a wide range of IPL values and most subjects crawl in trot-like pattern and no-limb-pairing pattern (IPL > 0.15) at low speed. When the crawling speed changed from 1 to 2 km/h, the inter-limb coordination variation was small. However, subjects showed a preference for trot-like crawling and pace-like crawling rather than no limb pairing when treadmill speed increased to a relatively high speed (≥2 km/h). And those subjects who used no-limb-pairing pattern could not keep pace with the treadmill and drop out from the belt. Only two subjects successfully kept pace with the treadmill at the speed of 2.5 km/h in no-limb-pairing pattern.

**Figure 6:**
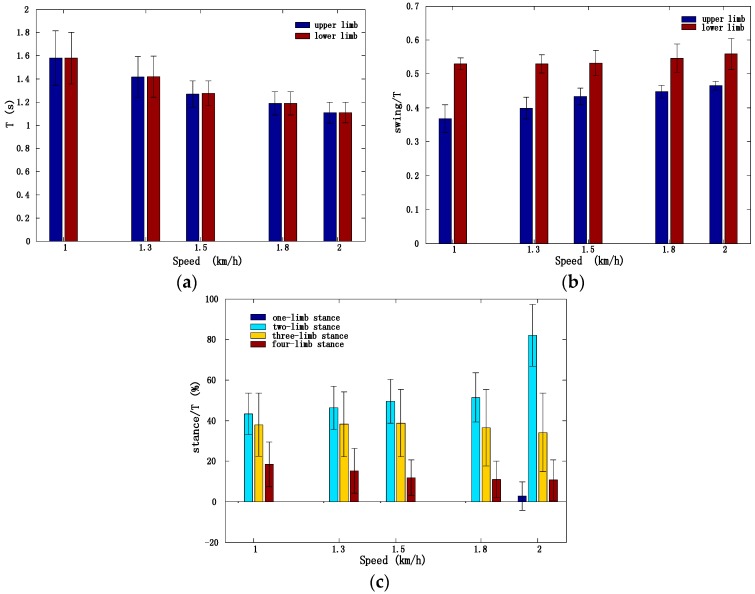
Crawling parameters at different speeds (from all 30 subjects, Mean ± SD). (**a**) Crawling cycle duration; (**b**) The proportion of swing phase; (**c**) The proportion of multi-limb stance phase (different number of limbs stay in stance phase at same time).

Figure 6 illustrates the variations in the cycle duration, the proportion of limb stance phase (swing duration versus crawling cycle duration), and the proportion of multi-limb stance phase (versus crawling cycle duration) at different speeds. As expected, the crawling cycle duration decreased as the crawling speed increased (Figure 6a). The proportion of single limb swing phase increased (Figure 6b) with the crawling speed, and upper limbs showed a shorter proportion of swing phase than lower limbs. Figure 6c shows that when crawling speed increased, the proportion of two-limb stance phase increased, the proportions of three-limb stance phase and four-limb stance phase decreased, and one-limb stance phase appeared at very high speed.

### 3.2. Motion Intensity, Stability, and Complexity Variations of Trunk under Different Speeds

**Figure 7:**
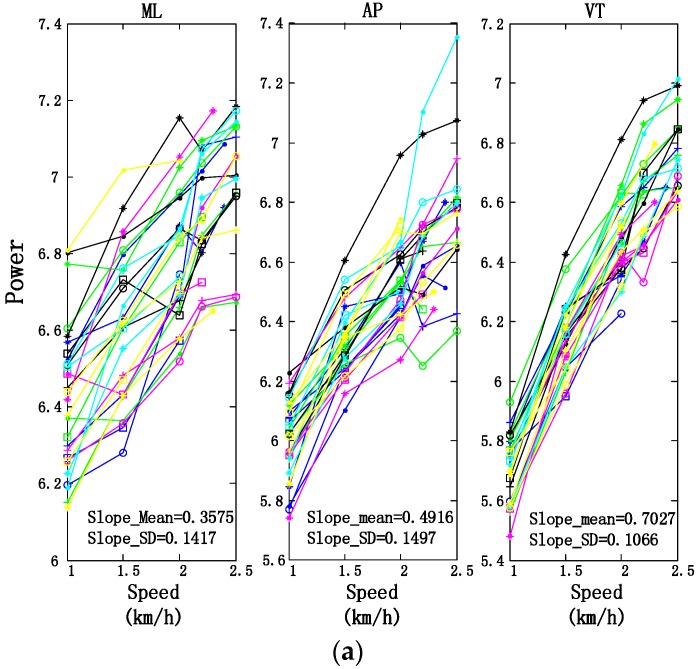

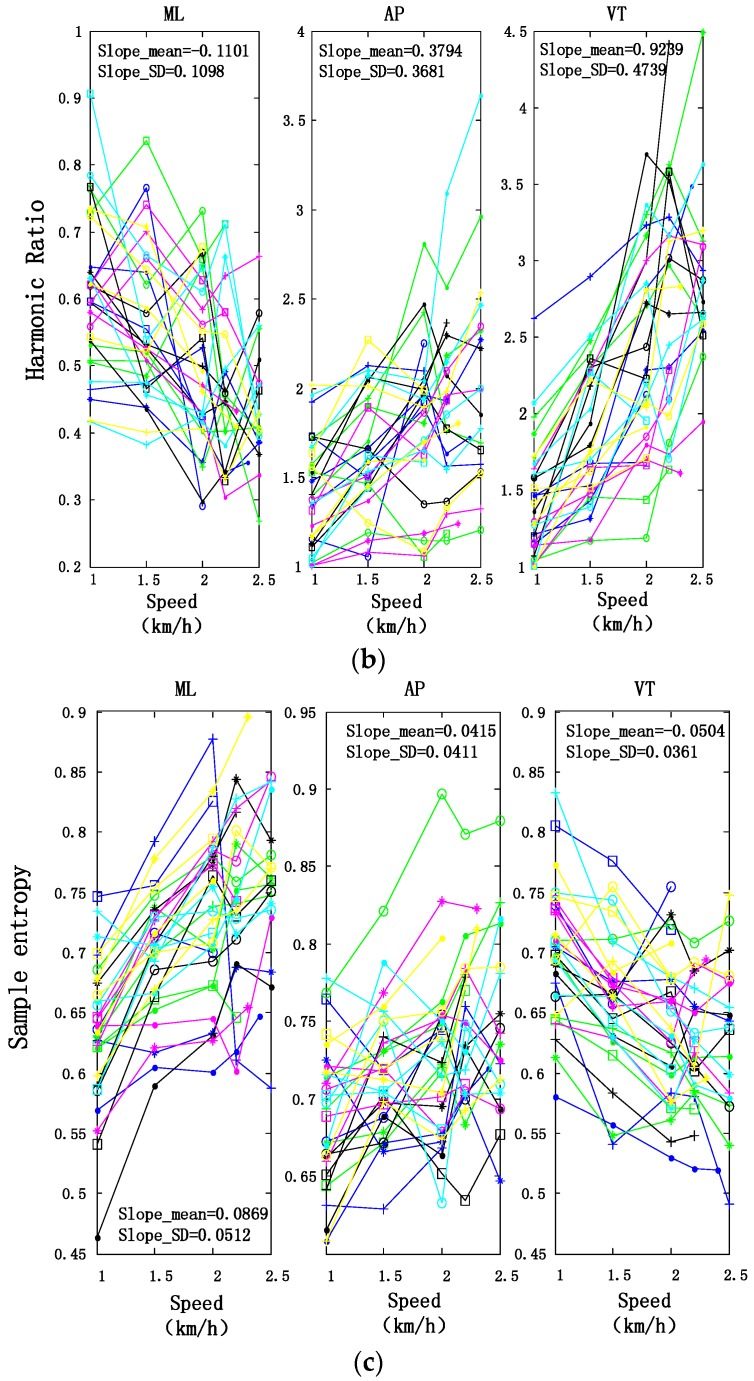
Trunk movement parameters of 30 subjects in ML, AP, VT orientation during crawling at different speeds. (**a**) Power; (**b**) Harmonic Ratio; (**c**) Sample entropy.

Power, harmonic ratio, and sample entropy of the waist acceleration signals were used to depict the intensity, stability and complexity of trunk movements respectively. Figure 7 presents these parameters of 30 subjects during crawling at different speeds. “Slope_Mean” means the average slope of the fitting lines for all subjects, and “Slope_SD” means the standard deviation of the slopes of these fitting lines. As shown in Figure 7a, power parameter increased with crawling speed at all 3 orientations and the average slope in VT orientation was the highest, which describes that trunk movement became more and more intense with the speed increased and most power changes occurred in the VT orientation. Figure 7b shows that harmonic ratio in ML orientation decreased, but values in AP and VT orientations increased with crawling speed, which means that trunk movement becomes less smooth in ML orientation, while smoother and more repeatable in VT and AP orientations. Like power, the highest slope of harmonic ratios was obtained at the VT orientation. In Figure 7c, the complexity of trunk movement shows an increasing trend with crawling speed in ML orientation and AP orientation, a decreasing trend in VT orientation, which means that trunk movement becomes more complex in ML and AP orientations but less complex in VT orientation with crawling speeds. Sample entropy of trunks show an increasing trend with crawling speed in ML orientation and a decreasing trend in VT orientation, which is opposite to the harmonic ratio. That is reasonable because entropy is a parameter to describe the complexity of time series by calculating the rate of new information. The movement is smoother and more regular, thus the rate of new information is lower.

### 3.3. Trunk Motion Intensity, Stability and Complexity Parameters Comparison between Three Types of Inter-Limb Coordination Patterns under Different Crawling Speeds

Power and sample entropy were also used to analyze the intensity and complexity of limbs movements in this study. According to H. John Yack and Roseanne C. Berger [26], harmonic ratio was used to judge the smoothness by measuring the repeatability of the biphasic pattern. Trunk movement during crawling can be seen as biphasic pattern because the trunk moved from side to side in a crawling cycle. However, the limbs kept swinging or standing in a crawling cycle, which resulted in a single-phase mode of the ACC signal. Thus, harmonic ratio of limbs movements was not analyzed. Only the power and sample entropy of left limbs was analyzed because the movements of left limbs and right limbs were symmetrical during crawling. As shown in Table 1, the power of trunk, upper limb, and lower limb all increased with crawling speed. The rising slopes of trunk power were higher than those of limbs, and upper limbs were higher than lower limbs. This result means that crawling speed has the most significant effect on trunk intensity followed by upper limbs and lower limbs. Different from trunk movement, sample entropy of limb movement increased with crawling speed in ML, AP, and VT orientations. The changes of the complexity in the lower limbs are higher than those in the upper limb in the ML and AP directions. In addition, the complexity of upper limb activity in VT direction was most affected by crawling speed.

**Figure 8:**
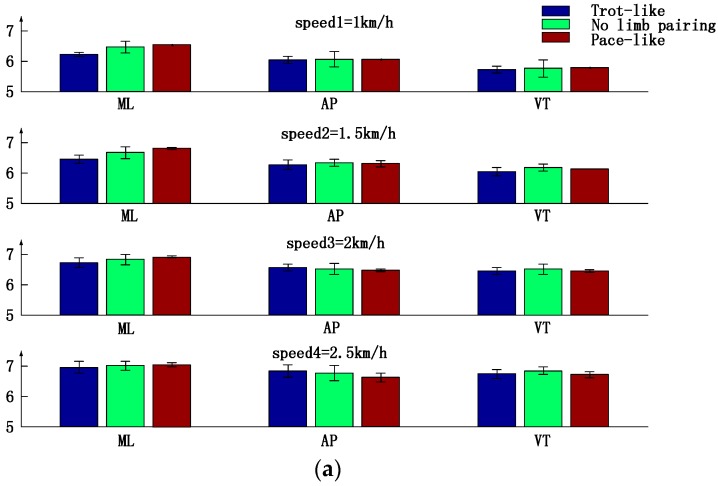

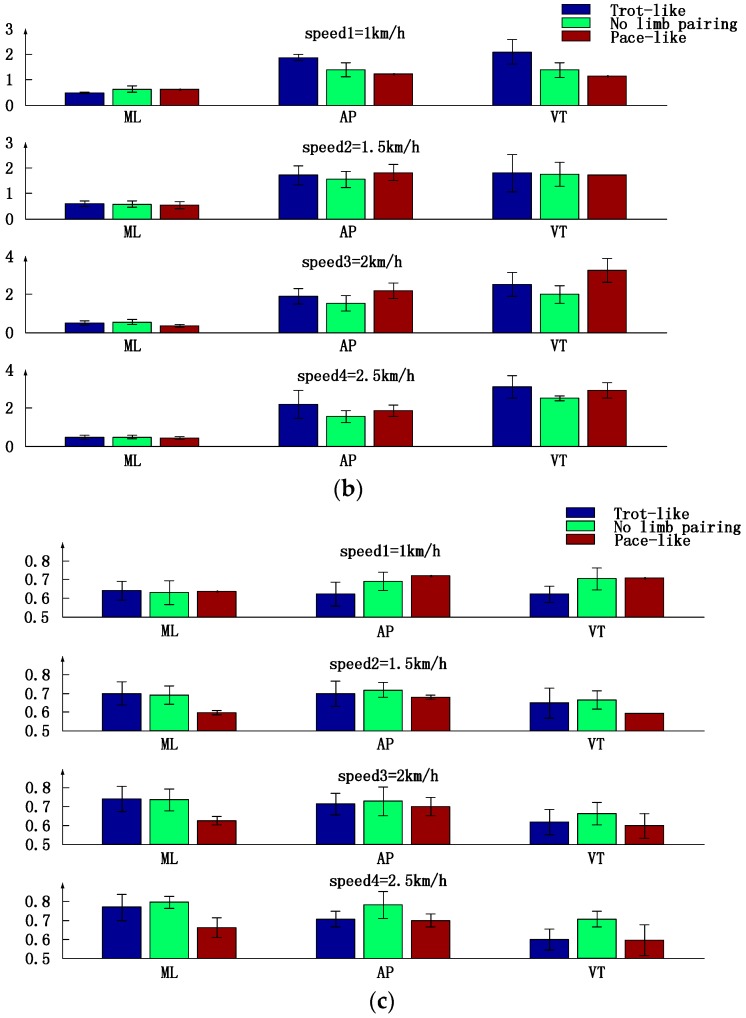
Trunk motion parameters comparison between three types of inter-limb coordination patterns under different crawling speeds. (**a**) Power; (**b**) Harmonic ratio; (**c**) Sample entropy.

Figure 8 compares trunk motion parameters between subjects in different inter-limb coordination patterns. As shown in Figure 8a, in any orientation, power shows slight differences between three inter-limb coordination patterns at the same speed, meaning that trunk movement intensity has no significant differences between subjects in different inter-limb coordination patterns. In Figure 8b, trunk harmonic ratios are less than 1 in ML orientation and greater than 1 in AP and VT orientations. At low speed (1 km/h), trot-like crawling has the highest harmonic ratios in AP and VT orientation, while pace-like crawling has the lowest in AP and VT orientations. As the speed increases, harmonic ratio of pace-like type grows rapidly, and the gap between the three patterns decreases. When it comes to high speed (speed = 2 km/h and 2.5 km/h), harmonic ratios of pace-like type are close to or higher than those of trot-like type. Meanwhile, no limb pairing type shows the lowest harmonic ratios in AP and VT orientations at high speed. According to Figure 8c, there is no significant difference in sample entropy between trot-like type and no limb pairing type in ML orientation, but pace-like type shows lower sample entropy than the other two types at high speeds. In AP orientation, trot-like type shows the lowest sample entropy and the pace-like type shows the highest sample entropy at low speed (speed = 1 km/h). With the increase of the speed, the gap between the three types decreases. When it comes to 2.5 km/h, no limb pairing type shows higher sample entropy than other two types. In VT orientation, trot-like type has the lowest sample entropy, and the pace-like type shows equal entropy with no limb pairing type at low speed (speed = 1 km/h). When it comes to 2.5 km/h, no limb pairing type has the highest sample entropy.

## 4. Discussion

Previous studies observed that human adults showed flexibility in inter-limb coordination patterns under different crawling speeds, but there was a weak relationship between speeds and inter-limb coordination patterns, as the selection of coordination patterns didn’t show a linear or consistent relationship with the value of speeds [6]. In this study, most human adults preferred trot-like and no limb pairing patterns at low speed, but trot-like and pace-like patterns when crawling speed increased to a relatively high value. As crawling speed increased, crawling cycle duration decreased, and the percentage of swing time increased. Cycle duration did not show difference between the upper and lower limbs, and swing duration was found consistently shorter in the upper limbs compared with the lower limbs. These results are consistent with those in related works [3,21,22]. Additionally, we observed that when crawling speed increased, the proportion of two-limb stance duration increased, the proportions of three-limb stance duration and four-limb stance duration decreased, and one-limb stance appeared at a very high speed.

Little is known about the reason for the variety of inter-limb coordination in four-limb locomotion, and the speculations about the inter-limb coordination pattern change caused by changing speeds have not been confirmed yet with enough evidence. In order to reveal the underlying mechanism of the inter-limb coordination change, the motion stability, intensity, and complexity of movements of trunk and limbs during crawling were also explored in this study. Power, harmonic ratio, and sample entropy of acceleration signals were used to depict the motion intensity, stability, and complexity respectively, while ipsilateral phase lag was used to depict inter-limb coordination pattern. With the increase of speed, the trunk movement was found to be more intense in all three orientations, more stable and regular in AP and VT orientations, while less regular and more random and complex in ML orientation. As discussed in related studies of walking gait, control of ML orientation motion could be thought to be under continuous feedback control allowing step-to-step adjustments for effective balance control [27,28]. Similar to walking gait, we speculate that the CNS keeps the stability of trunk in AP and VT orientations during crawling by adjusting the movement in ML orientation step by step. In this study, the average slope of trunk power was higher than the slopes of upper and lower limbs in three orientations. This result demonstrates that the change of crawling speeds has more significant effect on amplitude and frequency of trunk movements than limbs. Besides, different from trunk movements, the complexity of limb movement increased with crawling speed in all three orientations. The differences in motion intensity and complexity between trunk and limbs can be attributed to the fact that limbs, which have more degrees of freedom than trunk, are more flexible.

It is noteworthy that there is some relationship between inter-limb coordination pattern choice and the motion’s stability and complexity of trunk. At low speed (1 km/h), trot-like groups have the highest sample entropy values as well as the lowest values in VT and AP orientations among three crawling patterns. This result means that trot-like gait is the most stable and regular crawling pattern at low speed. From previous studies [2,6,9], it is well-known that the IPL value of healthy infants always distributed around 50%, which means that most infants tend to use a trot-like inter-limb coordination pattern. Meanwhile, mechanical factors including midline obstruction, limb length and unweighted crawling seem to have little influence on trot-like coordination pattern of infants. Patrick concluded the reason is the immaturity of infants’ nervous system [6]. Based on the results in this study, most adults choose trot-like coordination pattern when crawling at low speeds, perhaps because trot-like is the most stable pattern to keep themselves safe and maintain continuous motion. Also, at high speed, no limb pairing group shows the lowest stability and the greatest complexity in AP and VT orientations, which means no limb pairing is the least stable among three types. This phenomenon could explain why subjects tend to avoid no limb pairing pattern when speed is over 2 km/h, no matter which coordination type they used at low speeds. So, it is speculated that CNS could choose stable inter-limb coordination pattern to keep body safe and avoid the tumble.

Besides, crawling inter-limb coordination can also be understood from the perspective of motor control, which was defined to explore how the nervous system interacts with other body parts and the environment to produce purposeful movements [32]. First, motor abundance gives a concept that, for any given task, there are many functionally equivalent motor solutions [32,33]. These equivalent motor solutions are synthetically determined by the difficulty of the motor task, the ability of person and the factors of environment. Subjects could gain a balance in energy consumption, movement efficiency, and biomechanical constraints by choosing their motor solutions. In this study, the existence of several inter-limb coordination patterns during crawling could be considered as a phenomenon of motor abundance, and the inter-limb coordination pattern choices were affected synthetically by factors such as crawling speeds and individual ability. 

Second, John P Scholz introduced the dynamic pattern theory of movement coordination [34]. When some parameter reaches a critical value, the system may exhibit a transition to a new or different pattern of coordination. For example, a horse had a transition from trotting to galloping when it was forced to increase velocity of locomotion, and the relative phase of hands motion transited from 180° to 0° with motion speed increased, as the increasing frequency of movement led to loss of stability, so a pattern transition occurred at a critical value [34]. Dynamic pattern theory suggested that stability may be one of the reasons for the change of the movement pattern, and the new pattern emerged as a result of CNS interacted with environment and anatomical constraints. That is why patients like hemiparesis, who had some changes in body structure, usually have special motor patterns [35,36]. 

Some other research also provided insightful information about the dynamic pattern theory to explain the relationship between coordination pattern transition and central nervous system control over stability. Holt et al. investigated the preferred walking frequency of human, and they found that the preferred frequency was related to the energetic cost and stability [37]. Sides and Wilson investigated the intra-limb coordination in cycling with changing cadence and work rate, and they found that the stable coordination pattern would be maintained, which support the dynamic pattern theory [38]. Duncan et al. explained that participants would change posture when they performed a stationary standing task on a simulated ship at sea to reach dynamic stability and keep balance [39]. Based on the results in this study, it can be speculated that the spontaneous inter-limb coordination transition during crawling, from no-limb-pairing to trot-like or pace-like patterns when the speed increased to a critical value, should be caused by the loss of stability. This conclusion accords with the dynamic pattern theory. 

Third, trot-like pattern and pace-like pattern have the unity in diagonal limbs or ipsilateral limbs. Pairs of limb moving together seem easier to control than no limb pairing, which is a pattern with four limbs moving in turn. As the task difficulty increased with crawling speed, subjects prefer to select easy way to finish crawling task.

The research results of this study on the inter-limb coordination pattern choice during crawling from the stability, intensity, and complexity of trunk and limbs shed lights on the underlying motor control strategy of the human CNS and have important significance in the fields such as clinical diagnosis, rehabilitation engineering and kinematics research. We will try to explore the abnormal control strategy of crawling motion of children with cerebral palsy in future work. 

## 5. Conclusions

Taking human adults and hands-knees crawling as research objects, this study investigated the inter-limb coordination pattern choice under different speeds from the aspect of the motion stability, intensity, and complexity of trunk and limbs. The research results demonstrate that some relationship exists between inter-limb coordination pattern choice and the stability and complexity of trunk movement, which could explain why subjects tend to avoid no limb pairing pattern at high speeds no matter which coordination type they used at low speeds. The research result also provides evidence that CNS choose stable inter-limb coordination pattern to keep the body safe and avoid tumbling.

## Figures and Tables

**Table 1:** Comparison between trunk and limbs in motion intensity and complexity changes with crawling speed.

		Slopes of Fitting Lines (Mean ± SD)		
		ML	AP	VT
Power	Trunk	0.3575 ± 0.1417	0.4916 ± 0.1497	0.7027 ± 0.1067
	Upper limbs	0.3092 ± 0.1293	0.3992 ± 0.1170	0.4283 ± 0.1246
	Lower limbs	0.2884 ± 0.1936	0.2019 ± 0.1752	0.1191 ± 0.0438
SE	Trunk	0.0869 ± 0.0512	0.0415 ± 0.0411	−0.0504 ± 0.0361
	Upper limbs	0.0467 ± 0.0584	0.0202 ± 0.0645	0.1586 ± 0.0794
	Lower limbs	0.0735 ± 0.0540	0.0783 ± 0.0858	0.0914 ± 0.0629
